# Experience, Judgment and Luck

**Published:** 1899-09

**Authors:** Nelson W. Wilson

**Affiliations:** Buffalo, N. Y.; Attending obstetrician St. Mary’s Infant Asylum and Maternity Hospital


### EXPERIENCE, JUDGMENT AND LUCK.

### ILLUSTRATED BY A COMBINATION OF GONORRHEA AND PREGNANCY AND TWO UNUSUAL ABORTIONS.

By NELSON W. WILSON, M. D., Buffalo, N. Y.
Attending obstetrician St. Mary's Infant Asylum and Maternity Hospital.

A RECENT writer in designating the qualities which should be possessed by a successful physician, named an unfailing nerve, exquisite good nature and ready resource. To these might have been added experience, judgment and luck. They do not teach any of these in the medical colleges of today. One gets excellent instruction; one gets quizzed, and one gets the benefit of a teacher's experience, provided the teacher has that happy little trick—too rare unfortunately—of being able to give a parcel of raw-material students the good of his experience. But experience comes with practice; judgment comes with experience and luck comes sometimes like a visitation of Providence, masquerading as experience, and very often giving judgment and experience, both, the very healthiest kind of help when a lift is most needed.

When a new doctor begins practice he takes his first lesson in experience—judgment follows as a matter of course. If the Lord is with him, and his patient has fighting powers, his judgment is apt to be good. In other words, he has luck. Three cases will illustrate the triple title of this article.

In the days of the first flush of college weaning, when the faculty had signed themselves down on my diploma, and the great State of New York, through the regents, had officially certified to my ability to practise medicine and surgery, and given me a legal right to charge for my services, I was called to see a young woman who had come to Buffalo from the Queen's colony, across the river, full of shame, and a story of wrong. She had all the preliminary requirements for membership in a mothers' congress, save a marriage certificate and a ring. Being naturally sympathetic and anxious to show my interest in the fortunes and misfortunes of my patients, I listened to her long story of how it all happened.

Still, as a sort of reward for my sympathy and services to be rendered, I was informed that the gentleman who was at fault in the matter was willing and ready—nay, anxious—to pay all expenses of her illness. This and the fact that I was practising with all the earnestness, if not the ease, of a "real paid doctor, who carries a bag," enthused me. How I discovered that the young lady was also suffering with gonorrhea is quite as long, and quite as sad as the story of her life. Truly, the gentleman, secure under the protecting folds of the red cross of St. George, was lavish with his favors. I cannot truthfully say that he killed two birds with one stone, but it seemed, figuratively speaking, to amount to that.

At any rate, I went to work. Twice a day I visited that woman for days and days and days; I irrigated her carefully and conscientiously, and happy was the day when the discharge had ceased and she was rid of one trouble, with the other looming up in the near future.

I saw her, then, every few days after that for a few weeks, sympathising with her, cheering her and looking wise. And one morning I called and found that she was free from trouble and that her baby had been born in the early morning hours, without any discomfort to speak of, and that a neighboring physician had been called in after the birth of the baby and had taken charge of the case. While I was there he came in. He was an oldish man, with a supernaturally wise look and ragged whiskers. *En passant,* always get to windward of a supernaturally wise-looking man who wears his whiskers ragged. He had assumed charge, and he had a snap lock on that case. He could not relinquish the care of that baby, nor that woman. He always "saw a labor case through," he said. And he did. Oh, yes, I would be recompensed for my share of the work.

He saw the case through and the gentleman in Canada paid $40 good United States dollars to my friend with the supernaturally wise look and the ragged whiskers. I was, to use a racing term among the "also rans." I felt for a long time like that "fore and aft" regiment Kipling wrote about, which was stalked and potted from out the dark by the heathenish, knife wielding Paythans.

That is experience.

---

"I've had four children, an' I'm not goin' to have any more, doctor, not if I can help it, for th' old man's got a bad habit of drink, and the rent comin' due every thirty days, an' me doin' a full day's washin' every day for six days out of seven, to keep the sheriff out of th' house. I'm near four months gone now, an' can you gimme somethin' to help me."

That was the opening of she whom I call my judgment patient. I used, in answering her, the words of an old practitioner, who had given me the benefit of his years of wisdom, in many a comfortable hour's talk.

"You want to get rid of it?" I asked.

"I do," she said.

"If you will do as I say, I will guarantee that you wont have any trouble. You go home, and live as you have been living. Take care of yourself. Don't overwork and in five or six months you will be all right."

She came of a quick-witted race and she saw the point. She was good-natured about it, too. Two weeks later, I was sent for. It was Sunday, at 11 o'clock in the morning. I found her in bed.

"It came," she said, pointing vaguely at the bed clothes. She turned back the coverings and there between her thighs, wrapped in a piece of sheet, lay a fetus, probably four months, dead and dry, the clothes stained and hardened, the fetus attached to the cord, which passed into the vagina.

"When?" I asked.

"Last night, about 8 o'clock," she answered. That dead fetus had been there fifteen hours. Then I was stricken with a dash of judgment.

I covered up the little corpse and sat down and wrote a note to my old doctor friend, asking him to come. He came at once. While awaiting him I gathered the history. The previous Tuesday "after a hard day's washing" she had pains. They continued all Tuesday night and Wednesday. They eased up Thursday; Friday there were more, and still more Saturday. Saturday afternoon at 6 o'clock she went to bed and at 8 o'clock the fetus had come away dead. It was attached. She waited an hour or so for the placenta. It not coming she had tugged on the cord, but could not budge it. All Saturday night and early Sunday morning she had at different times tugged at the cord and failing to get it clear, had sent for me. I had recently delivered a vi-para in the house where she lived. The case was perfectly normal and things went along with neatness and dispatch. This woman was present, and I presume she looked upon me as a sort of a boy wonder.

When my old doctor friend came, I had her repeat the story to him. He saw the condition of affairs and after cutting away the fetus, tried to clean out the uterus with his fingers. He could not and finally curetted her. She was in anything but good shape. She recovered finally, however, under my old doctor friend's care. She probably would not had I plunged ahead on my own responsibility, and then there would have been all sorts of ugly aspects to the case. I consider that my judgment center was working full time when I sent for assistance and a man of years and irreproachable standing. He found things as I found them and he assumed charge. It was a charity case and that made me feel content at his compliment: "You showed great judgment in sending for me in a case like this."

---

The luck case came to me recently with this history. Six weeks previously she had a miscarriage. She was employed on a lake vessel, and believing that everything had come away continued her duties. She flowed considerably for a week. Then the flow lessened, for a few days only to begin again. This kept up for four weeks. She had lost considerable blood, evidently, for she said she was very weak. She went to a doctor in one of the cities at which the boat stopped, and "he put pincers in and took out the after-birth." That was two weeks previous to her visit to me, and she had been flowing most of the time since. She was very anemic and weak. Her legs was edematous and she was, as she expressed it "not a bit of good for anything."

I found the cervix dilated sufficiently to admit my index finger, and I felt and removed a piece of placenta the size of a silver dollar. The uterus was thoroughly cleaned out and irrigated. Her urine was examined and found to be albuminous. She was instructed to rest, was put on Basham's mixture and strychnine and she sailed away in her boat on its next trip out, a few days later, her duties being assumed by another woman. When the vessel touched Buffalo on its return trip I saw her. She reported that there had not been any flowing whatever, her urine had cleared up, the edema was about gone, and her lips was getting along toward the natural color. She was feeling better, too, and stronger:

If there is an element in this case more predominant than another, it is luck. Here was a woman who had every opportunity to enter upon a long and a dangerous siege of sepsis. I don't pretend to figure out how she escaped. I only know that she did, and I attribute it to luck.

131 FRANKLIN STREET.

